# Utilizing Plant Synthetic Biology to Improve Human Health and Wellness

**DOI:** 10.3389/fpls.2021.691462

**Published:** 2021-08-24

**Authors:** Collin R. Barnum, Benjamin J. Endelman, Patrick M. Shih

**Affiliations:** ^1^Department of Plant Biology, University of California, Davis, Davis, CA, United States; ^2^Lawrence Berkeley National Laboratory, Environmental Genomics and Systems Biology Division, Berkeley, CA, United States; ^3^Feedstocks Division, Joint BioEnergy Institute, Emeryville, CA, United States; ^4^Genome Center, University of California, Davis, Davis, CA, United States; ^5^Department of Plant and Microbial Biology, University of California, Berkeley, Berkeley, CA, United States; ^6^Innovative Genomics Institute, University of California, Berkeley, Berkeley, CA, United States

**Keywords:** plant synthetic biology, natural products, nutraceuticals, pharmaceuticals, human health, nutrition

## Abstract

Plants offer a vast source of bioactive chemicals with the potential to improve human health through the prevention and treatment of disease. However, many potential therapeutics are produced in small amounts or in species that are difficult to cultivate. The rapidly evolving field of plant synthetic biology provides tools to capitalize on the inventive chemistry of plants by transferring metabolic pathways for therapeutics into far more tenable plants, increasing our ability to produce complex pharmaceuticals in well-studied plant systems. Plant synthetic biology also provides methods to enhance the ability to fortify crops with nutrients and nutraceuticals. In this review, we discuss (1) the potential of plant synthetic biology to improve human health by generating plants that produce pharmaceuticals, nutrients, and nutraceuticals and (2) the technological challenges hindering our ability to generate plants producing health-promoting small molecules.

## Introduction

Plants are some of the greatest chemists on our planet. They offer a vast, barely tapped repository of potentially bioactive compounds, with current estimates predicting over 200,000 unique specialized metabolites across the plant kingdom (Fang et al., 2019). Many of these metabolites act as therapeutic phytochemicals and essential nutrients in humans, making plants an invaluable source of bioactive compounds. However, barriers, such as the lack of access to healthy foods, limit the availability of these essential nutrients for human consumption (Hendrickson et al., 2006). Plants also produce a wealth of therapeutic phytochemicals, both pharmaceuticals and nutraceuticals (bioactive natural health-enhancing compounds in food), that are difficult to chemically synthesize, leaving consumption of medicinal plants or plant extracts as the sole source of these important chemicals (Nogueira et al., 2018). Additionally, many important phytochemicals are expressed in plants that are difficult to cultivate or produce insignificant amounts of the desired phytochemical (Nogueira et al., 2018). With an increasing global population, new tools are needed to provide highly nutritive foods and low-cost therapeutics to a larger populace.

**FIGURE 1:**
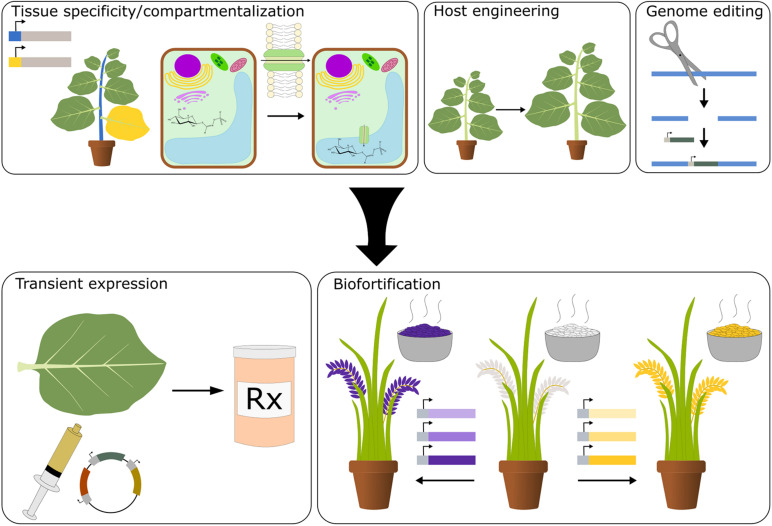
Plants as a platform for synthetic biology. Improvements in tissue-specific promoters, tools for compartmentalization, plant host engineering, and genome editing will aid in the development of plants as a platform for the production of health promoting small molecules and the enrichment of crops with nutritive chemicals.

Synthetic biology is a proven approach to reconstitute metabolic pathways for the production of valuable chemicals; so far, microbes have been the production platform of choice. While an extremely robust synthetic biology platform, microbes are unicellular organisms, hindering the reconstitution of certain complex plant metabolic pathways that normally take place in multiple cellular compartments and tissue types (Zurbriggen et al., 2012). Additionally, many plant enzymes do not function properly in microbial systems (Kotopka et al., 2018). Microbial production also requires the use of expensive, large-scale fermenters and the extraction of metabolites produced, incurring significant costs. Plants are ideally suited for the production of plant natural products as they natively host many of the biosynthetic pathways and produce essential precursor molecules required using light and water. Additionally, plants have a variety of tissues and organelles, allowing for compartmentalization of pathways and toxic intermediates. As humanity’s main source of food, plants are a unique yet practical vehicle for the production and delivery of essential nutrients and nutraceuticals (Figure 1). While plants present an ideal platform, there are still technological bottlenecks hampering our ability to optimize the production and accumulation of specific nutrients and phytochemicals. This review will examine the past successes and future challenges of plant synthetic biology in producing valuable therapeutics and enhancing the nutritive capacity of food crops. Additionally, the strengths and weaknesses of plants as a production platform will be addressed.

## Plant Product Development

Plants and the compounds they produce are some of the most important natural resources we have. From the food we eat to many of the medicines we take, plants provide us with a diverse selection of bioactive compounds capable of improving human health. A major goal of plant synthetic biology is to identify biosynthetic pathways for bioactive compounds and transfer them to other plant species to provide alternative sources of these compounds. In this section, we focus on the progress of pharmaceutical compound biosynthetic pathway discovery, efforts for enhancing the nutritive value of plants, and exploration of nutraceuticals for better health and wellness.

### Chemistry of Plants for Compound Discovery—Pharmaceuticals

Plant remedies have provided insight into functional molecules that have drastically improved human healthcare, such as the revolutionization in pain management following the use of opiates from opium poppy and the discovery of the active ingredient, salicin, in willow bark which enabled the synthesis of aspirin by the acetylation of plant derived salicylic acid (Desborough and Keeling, 2017; Labanca et al., 2018). However, as was the case with aspirin, it can be difficult to pinpoint the specific metabolite, or combination of metabolites, that result in observed beneficial effects from the entirety of a plant’s metabolome. With the major advances in modern analytical chemistry tools, it has become easier to identify and characterize specific metabolites from any given organism (González-Riano et al., 2020). From this collection of metabolites, specific compounds can be isolated and tested for bioactivity and efficacy against many chronic and deadly diseases. However, knowing the identity of the compound is only the start, as many of these active compounds are found in uncultivated plant varieties or in low abundance therein, impeding the large-scale production necessary for pharmaceutical use (Atanasov et al., 2015).

Plant synthetic biology seeks to circumvent some of these problems through heterologous expression of bioactive compounds for large-scale production and extraction. The first step needed to achieve this goal is to fully characterize the biosynthetic pathway for the compound of interest. Understanding the reactions between pathway intermediates and determining the required parts in a pathway based on standard classes of enzymes can help to fill in the gaps of many incomplete biosynthetic pathways (Anarat-Cappillino and Sattely, 2014). Additionally, modern sequencing technologies have advanced to the point where whole genome sequencing and transcriptomics in conjunction with co-expression analysis, phylogenomics, gene clustering, and genome-wide association studies of non-model species have become accessible (Jacobowitz and Weng, 2020). These techniques enable a route to the rapid discovery of all the parts in complex biosynthetic pathways, which enables heterologous expression of pathways for compound production. A notable example of this is the discovery of the pathway for the anti-inflammatory drug, colchicine, from *Gloriosa superba* (Nett et al., 2020). In this work, the authors used previously generated RNA-seq data from multiple species, generated a new RNA-seq dataset, and used metabolomics to determine eight essential genes in the pathway as well as reconstitute a 16-gene pathway with transient expression in *Nicotiana benthamiana* to produce the colchicine precursor, N-formyldemecolcine. With these tools in hand, it is time for plant synthetic biology to develop a library of parts and pathways to not only produce natural bioactive compounds for better human health but also new-to-nature compounds which could revolutionize the way we think about wellness.

Other examples of heterologous expression of phytochemical pathways have shown it is possible to develop plants into pharmaceutical production platforms. *Crocosmia* spp. is an ornamental plant that was found to produce Montbretin A (MbA), a potent inhibitor of human pancreatic amylase, which is a promising treatment for type II diabetes awaiting clinical validation (Tarling et al., 2008). However, MbA is only produced in the corms of *Croscosmia* spp. in low amounts. Corms are the small vegetative reproductive tissue of the plant, making the propagation and extraction of MbA difficult. Thus, the limitations of *Crocosimia* spp. make a heterologous host a better suited system for MbA production. The full MbA pathway was recently discovered (Irmisch et al., 2019) and its heterologous expression in leaf tissue of *N. benthamiana* resulted in a measurable but low yield, displaying the need for pathway and potentially host optimization for large-scale production. Potential mechanisms for effective pathway optimization include enhancing precursor molecule levels or relaxation of bottleneck steps within the pathway. Efforts in optimizing metabolic flux *in planta* have been demonstrated with other plant natural products. For example, etoposide is a chemotherapy drug whose precursor previously could only be isolated from the endangered mayapple plant, *Sinopodophyllum hexandrum*. Optimization of the production of the etoposide precursor, (−)-deoxypodophyllotoxin, was improved by two orders of magnitude through transient expression of eight genes required to produce coniferyl alcohol, allowing for purification of milligram levels (Schultz et al., 2019).

Traditional medicinal plants are another source of pharmacologically important compounds, with several compounds having fully elucidated biosynthetic pathways. Salidroside, isolated from the *Rhodiola* genus, has a long history of use due to its potential, albeit understudied, benefits for mood stabilization, fatigue, and prevention of cardiovascular disease and cancer (Zhang et al., 2021). Unfortunately, because of its suggested benefits, some species of *Rhodiola* are being harvested to near extinction from their native habitats. The recent discovery of its three-enzyme biosynthetic pathway using transcriptomics and metabolomics has enabled the production of salidroside by heterologous expression in *N. benthamiana*, providing a scalable alternative to extraction from *Rhodiola* (Torrens-Spence et al., 2018). From another medicinal plant, *Catharanthus roseus*, the final steps of the complicated, thirty-one enzyme biosynthetic pathway of vinblastine, an important drug in the treatment of leukemia and lymphoma, have been discovered using RNA-seq to identify the genes and transient expression in *N. benthamiana* to validate vinblastine production (Caputi et al., 2018; Qu et al., 2018). The heterologous expression of this pathway could provide an alternative production platform for the synthesis of this important pharmaceutical. Even with these discoveries, there are many biosynthetic pathways of important medicinal compounds that are not fully understood, such as tentative antivirals found in traditional Chinese medicine (Xian et al., 2020). Currently, some of these traditional medicines are undergoing FDA clinical trials to be developed into direct treatment or combinatorial supplements with existing approved drug treatments (Li and Weng, 2017). With continued progress, there will be a more comprehensive library of pharmacologically relevant phytochemical pathways to draw upon for the treatment of any type of disease.

With a greater understanding of the biosynthetic principles behind natural product synthesis, synthetic biologists are producing new-to-nature molecules by utilizing well-characterized precursors in combination with modifying enzymes from multiple plant sources. Terpenoids are ubiquitous across the plant kingdom and this diverse class of molecules serves many functions for plants, including but not limited to defense and stress response. Researchers have demonstrated that heterologous expression of the triterpene, ß-amyrin, with combinations of CYP450 enzymes can produce more than a dozen natural saponins as well as new-to-nature products on a gram scale (Reed et al., 2017). These compounds were tested for anti-proliferative and anti-inflammatory effects and demonstrated some effectiveness, illustrating the potential pharmacological efficacy of new-to-nature compounds being produced in plants at a large scale. Other evidence shows that the addition of plant and bacterial enzymes into a plant-specific pathway can also enhance the ability of plants to generate new-to-nature compounds. A recent example of this is the production of novel crucifalexins and halogenated derivatives of brassinin by transiently expressing halogenases and various CYP79 genes with different specificities (Calgaro-Kozina et al., 2020). The authors also demonstrated that the antifungal nature of these new compounds was enhanced compared to commercially available products, showing an alternative approach to developing a suite of new compounds using the parts and pathways that are readily available.

### Biofortification of Essential Nutrients

One of the primary ways plant synthetic biology can aid in health and wellness is by alleviating malnutrition which is still prevalent across the entire world. Beyond the macromolecular necessities of food, an even greater portion of the world experiences micronutrient deficiencies—termed “hidden hunger”– especially in regard to vitamin A, iodine, zinc, and iron (Muthayya et al., 2013). A recent review (Malik and Maqbool, 2020) has an in-depth analysis of the types of deficiencies as well as some strategies that have been used to biofortify crops. The production of palatable crops that provide adequate amounts of macro- and micronutrients that can grow in diverse biomes is one of the cheapest and most practical ways to ameliorate malnutrition (Matsuura et al., 2018). Plant metabolic engineering has the potential to address many of these nutrient shortcomings through both the manipulation of existing pathways and the introduction of additional plant pathways.

Traditionally the enhancement of crop species has occurred through breeding practices which involves a series of genetic crosses and backcrosses to take a desirable trait from one variety of plant (such as a wild relative) into a commercial cultivar. This process requires multiple plant generations and can introduce undesirable effects due to tight gene linkage. These practices have been in use for thousands of years and have improved almost every crop that we eat today, yet the arduous nature of plant breeding has limited the varieties of crops that are actively cultivated for consumption. With the advent of gene-editing technologies, scientists can directly modify specific DNA sequences to produce desired changes in the plant genome within one generation. An example of this is in the improvement of ground cherry, a species closely related to tomato (Lemmon et al., 2018). In this work, the authors use the clustered regularly interspaced short palindromic repeats (CRISPR)–CRISPR-associated protein-9 nuclease (Cas9) (CRISPR–Cas9) system to generate null mutations in *Ppr-SP5G* and *SlCLV3* promoter region to impart desirable traits into this crop, such as flowering and fruit size. Additional examples of *de novo* domestication from wild tomatoes have shown the potential of nutritive enhancement along with cultivation improvement, retaining the disease and abiotic stress resistance of the wild varieties (Li T. et al., 2018; Zsögön et al., 2018). With the use of new tools to perform genetic modifications, such as CRISPR-Cas9, species of plants that are difficult to cultivate can be modified for use as crops, thereby diversifying available food options. This could in turn enhance the availability of nutrients that are limited throughout the world. Additionally, the advent of a CRISPR-Cas9 with less restriction on the genetic site of protein binding will allow for greater flexibility in the use of this tool (Ren et al., 2021). Gene editing further allows for the alteration and enhancement of specific endogenous biosynthetic pathways to produce biofortified crop species. Work has shown that silencing of lycopene ε-cyclase in the carotenoid pathway can enhance the natural levels of ß-carotene while reducing the α-carotene levels (Ma et al., 2011). Other examples show that the manipulation of plastid identity—such as the conversion of chloroplast to chromoplast—can enhance the overall carotenoid levels. One study converted chloroplasts to chromoplasts in tomato during early fruit development through the ectopic expression of the *Arabidopsis thaliana* ORANGE gene containing a SNP (*AtOR*^*His*^) (Yazdani et al., 2019). Another study converted chloroplasts to chromoplasts throughout the plant through the expression of plastid-targeted microbial phytoene synthase (*crtB*) in multiple plant species; however, the transgenic plants suffered from decreased photosynthetic efficiency in leaf tissue (Llorente et al., 2020). This highlights the importance of finding the balance between high titers and stable growth. Additionally, the levels of zeaxanthin, a carotenoid with potential benefits in eye health, have been increased by the elimination of zeaxanthin de-epoxidase in the algal species *Chlamydomonas reinhardtii* (Song et al., 2020). The enhancement of desirable carotenoids could easily be translated into higher plant species and much effort is being made for their use in non-model organisms (Zhang et al., 2019; Shan et al., 2020). There is also evidence that CRISPR-Cas9 modifications to upstream open reading frames (uORFs) can impact metabolite regulation across species, as vitamin C concentrations increased by 150% in *Arabidopsis* and lettuce by removing the regulatory effects of the associated uORF (Zhang et al., 2018).

While altering the levels of endogenously produced chemicals is effective, this approach is limited in the diversity of nutrients it can deliver. However, plant synthetic biology offers a means to bypass this limitation through the introduction of heterologous biosynthetic pathways. One of the first successes in this came with the production of golden rice, a ß-carotene enhanced rice grain that was generated through the addition of three genes (Ye et al., 2000). Since then, replacement of the daffodil phytoene synthase with a maize phytoene synthase has increased the titers of ß-carotene in golden rice 2 (Paine et al., 2005), providing greater nutritional benefits (Singh et al., 2017). Furthermore, the authors were able to not only add ß-carotene to rice grains but also increase the levels of iron and zinc with a single genetic insertion. This shows the potential of a biofortified staple crop to have an enhanced nutritive capacity suitable to meet multiple minimal daily requirements. Additional work has shown that the added ß-carotene in the rice grain can be further modified into astaxanthin and canthaxanthin, two antioxidants of interest, with one or two additional genes (BHY and BKT; Zhu et al., 2018).

Iron deficiency is one of the world’s leading micronutrient deficiencies (World Health Organization, 2013) and is responsible for around one million deaths annually. As such, iron has been a target of biofortification efforts over the last few decades. Early work showed that the overexpression of a root zinc transporter from *Arabidopsis* (AtZIP1) could not only increase the zinc concentration in grains, another significant deficiency, but it also increased the iron content for *Hordeum vulgare* (Ramesh et al., 2004). The addition of genes increasing nicotianamine, the nicotianamine-iron transporter and the iron storage protein ferritin was shown to have a concerted effect on rice seed iron concentration of more than fourfold (Masuda et al., 2012). Perhaps, with the combination of nutrient transport (reviewed in Yadav et al., 2021) and sequestration mechanisms in the grain or fruit, levels of many micronutrients could be enhanced.

Other vitamin deficiencies also pose major concerns to human health and plant synthetic biology efforts can alleviate some of these nutrient shortcomings. Folate is an essential nutrient needed for these biological processes in humans. Inadequate folate consumption is linked to cardiovascular disease, Alzheimer’s disease, and cancer, but it is especially important for pregnant women, as deficiencies can cause neural tube defects or megaloblastic anemia (Iyer and Tomar, 2009). Folate content in rice has been improved by 150-fold through the expression of a folate binding protein and a folylpolyglutamate synthase (Blancquaert et al., 2015), offering a means to increase folate consumption without dietary supplementation. This work also showed that the binding protein provided stability to folate so that it was maintained at a similar level even after months of storage, which is a concern for any crop that does not get eaten immediately after harvest. Vitamin B6 deficiency has been shown to be linked to cardiovascular disease, diabetes, neurological diseases, as well as nodding syndrome (a childhood disorder common in parts of Africa). Efforts to biofortify cassava, a staple vegetable crop in many parts of Africa, have been successful with the expression of the *Arabidopsis thaliana* genes PDX1.1 and PDX2, which are involved in *de novo* production of vitamin B6. Root-specific expression of PDX1.1 and PDX2 increased vitamin B6 and B6-glycoside concentrations by 4- and 14-fold, respectively, even after cooking (Li et al., 2015).

While biofortification of crops has taken great strides forward, there remain several considerations for effective implementation of this synthetic biology strategy. As more compounds are engineered into crops, it will be important that metabolic flux and regulation are not drastically altered, as this can have detrimental pleiotropic effects on the plant’s health. For example, the overproduction of γ-aminobutyric acid in tomatoes causes gross developmental defects (Li R. et al., 2018). Moreover, there will be clear challenges in transferring metabolic engineering strategies from one crop to another. There has been moderate success in this, such as with enhancing ß-carotene in golden bananas (Paul et al., 2017), but each crop is unique and requires experimentation to optimize production. As we continue to understand the intricacies of plant metabolism, these issues will be more easily addressed.

### Nutraceuticals and Disease Prevention

Nutraceuticals are defined as consumed bioactive compounds that are not essential for nutrition but offer other health benefits. These molecules are distinct from pharmacological compounds—though in some cases they do overlap—and do not require extraction and purification or semi-synthetic modifications before final dosage and distribution, as the source of nutraceuticals are the foods themselves. While these compounds are not essential for growth and development and many of their health benefits are still under investigation, their presence in our diet has the potential to improve human health.

One nutraceutical compound that has been of great interest is resveratrol. This naturally occurring compound, found in wine and chocolate among other plants, has been studied since the 1990s for its health benefits due to its association with the “French paradox,” the quandary of a low incidence of heart disease in a population that eats a high-fat diet. Many health benefits, including prevention of cardiovascular disease and treatment of diabetes, have been attributed to resveratrol and validated clinically, but its low concentration in native plants poses a challenge to extraction for medicinal use (Pannu and Bhatnagar, 2019). Additionally, consumption of purified resveratrol is mostly metabolized, greatly reducing its beneficial effect, but ingestion alongside other food-based phenolic compounds increases resveratrol levels found in blood serum. Therefore, the addition of resveratrol to foods could provide a more efficient means for delivery than purified supplements. Since all plants contain the precursor molecules for resveratrol, 4-coumaroyl-CoA and malonyl-CoA, only a single gene, stilbene synthase, is required to produce minor amounts of the metabolite in any plant. Optimization of precursor pathways would also be necessary to enhance nutraceutical levels in any given food (Jeandet et al., 2012).

Glucosinolates, and their hydrolysis products, isothiocyanates, are another family of compounds with great health potential that have been studied thoroughly (Neave et al., 2005; Lee et al., 2019; Yagishita et al., 2019). A recent review examined the vast diversity of these compounds, totaling 156 unique entries, emphasizing their identification (dating back to the first use of the nomenclature in 1961) and synthesis in plants (Blažević et al., 2020). This diversity can be broken down into three types of glucosinolates based on the amino acid precursor: aliphatic, indolic, or benzenic, with the vast diversity derived from secondary modifications to these basal categories. Over decades of study, biosynthetic pathways for each type have been elucidated (reviewed in Sønderby et al., 2010; Chhajed et al., 2020). Glucoraphanin is a glucosinolate found in high concentration in broccoli and has been a subject of intensive study due to the health benefits of it and its isothiocyanate, sulforaphane (reviewed in Yagishita et al., 2019). Various studies have displayed the promise of sulforaphane in treating diseases such as neurological disorders, diabetes, cancer, and cardiovascular disease with variable efficacy observed over a vast range of dosages and sources of the metabolite; however, further clinical validation is needed to verify these health benefits (Yagishita et al., 2019). Medicinal interest in glucoraphanin led to its successful production in *Nicotiana benthamiana* through transient expression, though only small amounts were produced (Mikkelsen et al., 2010; Crocoll et al., 2016). This was one of the first examples of a complex pathway being heterologously expressed in plants, and with some optimization, it could be stably introduced into a crop species for consumption.

The use of breeding practices that select plants for favorable agricultural traits can remove important nutrients from cultivated crops. This can also reduce some of the important nutraceutical compounds found in the foods that we eat. For example, there is a complete lack of iridoids, a class of monoterpenes suggested to benefit human health, in most cultivated varieties of blueberry on the market, while every wild species tested was abundant with them (Leisner et al., 2017). While this was an inadvertent consequence of breeding, it illustrates the need to investigate wild relatives of food crops to identify nutraceuticals lost during domestication. Using the tools of plant synthetic biology, we should be able to quickly and easily reintegrate the pathways lost into commercial cultivars without the need for the lengthy breeding process.

As we move forward in our efforts to produce advanced nutritive foods, it is important to examine the past uses of nutraceuticals in the prevention and treatment of disease. Traditional medicinal plants have seen renewed interest as sources for undiscovered bioactive compounds (World Health Organization, 2013). This highlights the need for additional compound and pathway discovery efforts. There are many other reviews that discuss the different types of specialized metabolites found in medicinal plant species (Mgbeahuruike et al., 2017; Saucedo-Pompa et al., 2018; Anand et al., 2019). Historically, medicinal plant extracts have been used as food additives, but introduction of their biosynthetic pathways into common food crops offers a means of increasing the availability of nutraceuticals to a broader market. In some cases, the biosynthetic pathways are known (see Yang et al., 2016 for an in-depth review) and efforts are being made to sequence more medicinal plant genomes in order to accelerate enzyme discovery in candidate organisms (Brose et al., 2021). Nevertheless, the major limiting step hampering efforts to engineer plant nutraceuticals is still the discovery of the enzymes involved in the biosynthesis of target plant natural products. Thus, new tools to streamline such research is of great interest to the larger plant synthetic biology field.

## Opportunities and Obstacles in Using Plants as a Production Platform

Plants have many attributes that make them an ideal platform for the production of plant natural products. They are autotrophic, can be grown at a large scale, contain various intracellular compartments and tissue types, are used as a nutrient delivery system, and retain similar cellular features to the product-producing plant that may be required for efficient biosynthesis of compounds. Together, this enables countless strategies for the production and administration of plant natural products (Figure 1). However, the state of plant biotechnology has imposed limitations on the utilization of plants. In this section, we focus on the strengths of plants as a production platform and the technologies needed to improve their efficacy.

### Scalability

Perhaps one of the greatest strengths of plants as a synthetic biology platform is their scalability. Large-scale plant production has been a focus of human society and technology since the first crops were cultivated. In addition to a long history of scalable plant cultivation for consumption, agriculture also offers a unique platform for the production and extraction of valuable therapeutic plant natural products. Opioids from *Papaver somniferum*, taxol from *Taxus brevifolia*, and QS-21 adjuvants from *Quillaja saponaria*, are several notable examples of therapeutic molecules that are produced and extracted from their native plant host (Jennings et al., 1992; Labanca et al., 2018; Qi and Fox, 2021). While many therapeutic chemicals can be extracted from their native producer, many species are difficult to cultivate or suffer from limited yields (Nogueira et al., 2018). Past efforts have focused on the production of nutrients and phytochemicals in microbes or with the use of chemical synthesis, but plants are an ideal platform for the production of small molecules at a large scale, as they are autotrophic and can be grown in non-axenic conditions like an open field.

There are two main approaches for the heterologous expression of a specialized metabolic pathway *in planta*: transient expression or expression in stable plant lines. Transient expression typically utilizes *Agrobacterium tumefaciens* to insert genes of interest into a host plant. This process allows for the expression and characterization of genes in 3–6 days as opposed to the months required to make stable plant lines (Pouvreau et al., 2018). Additionally, multiple strains of *Agrobacterium* harboring different genes can be co-infiltrated into host tissue, limiting the need for creating multi-gene constructs (Reed and Osbourn, 2018). These attributes have made it a valuable tool for *in planta* pathway discovery and protein production. Transient expression is usually conducted in a species of the genus *Nicotiana* but is typically only applied at small scales, limiting its viability as a production platform. Recent improvements by Reed and Osbourn (2018) have demonstrated the scalability and viability of transient expression for the production of phytochemicals. Through the use of full plant transient expression via vacuum infiltration, gram-scale quantities of various triterpenes were able to be produced and purified from *Nicotiana benthamiana* (Reed and Osbourn, 2018).

As well as being a production platform, transient expression permits the testing of gene construct efficacy prior to stable line generation; however, the efficiency of *Agrobacterium*-mediated transient expression is variable in different hosts (Zhang et al., 2020). This poses a limitation of *Agrobacterium* as a tool to screen genes in host plants. Additionally, transiently expressed pathways in *Nicotiana benthamiana* do not always function as expected in the desired host, limiting its use for screening constructs before generating stable lines (Fischer et al., 2013). One method to circumvent these issues is the use of protoplasts, calli, and cell suspension cultures. These cell types are suitable for the use of direct transformation methods such as electroporation, gene gun bombardment, and microinjection. However, generation of these cell lines is often inefficient depending on the species used, produces fragile cells, and may not behave as an intact plant would (Jores et al., 2020). A recent study utilized an industrially scalable delivery of self-replicating viral vectors to transform a variety of crop species, such as tomato, potato, and spinach, with successful alterations to reproduction, stature, and drought tolerance (Torti et al., 2021). This method could be further developed for the introduction of biosynthetic pathways for bioactive phytochemicals into single generations of crop species. Advancements in the host range and efficacy of transient expression will make plants a better platform for the production of plant natural products and the characterization of pathways in hosts before the generation of stable plant lines.

Transgenic plants can be grown in open fields, allowing plant natural products to be purified at large scales. However, the generation of stable plant lines is arduous, typically requiring the use of low-efficiency transgene delivery methods with *Agrobacterium* or particle bombardment, the generation of calli, the growth of the new, transgenic plant, and the confirmation that the transgene is properly inserted. This results in long regeneration times, a requirement for numerous generations of backcrossing, and limits the size and location of transgene insertions. A series of studies have begun to ameliorate this by creating transformation methods in maize that have increased the number of genotypes amenable to transformation, removed the need to generate calli, and utilized systems to remove selectable markers (Lowe et al., 2016, 2018; Wang et al., 2020). Additionally, improvements over the size of transgenes that can be inserted have been made. Dong et al. (2020) utilized CRISPR-Cas9 in rice to perform targeted insertion of the 5.2 kb carotenoid biosynthesis pathway in a genomic safe harbor to limit adverse insertion effects. This method also limits the segregation of transgenes in subsequent generations, potentially reducing the number of crosses needed. With simplification of breeding efforts, the production of nutraceuticals and phytochemicals in transgenic plants will be ready for market more rapidly by requiring less screening of transgenic plant lines for insertional effects. Another example utilized a gene stacking method called TransGene Stacking II to generate and insert a 31 kb cassette encoding an anthocyanin pathway into rice grain (Zhu et al., 2017). The utilization of gene stacking and targeted integration will enable long, complex pathways to be inserted with minimal adverse insertion effects (Shih et al., 2016). Together, these techniques have improved the feasibility of making stable plant lines expressing complex metabolic pathways for nutraceuticals and phytochemicals.

Many transformation techniques that generate stable transgenics require lengthy and expensive approval processes before becoming marketable (Skraly et al., 2018). The use of gene editing tools with high target specificity that can be removed from future generations is able to circumvent these approval processes. One example is the modification of soybeans through TALEN site-directed mutagenesis, wherein natural soybean oil contains high levels of linoleic acid, an undesirable compound increasing the risk of heart attacks (Haun et al., 2014). In this work, FAD2-1 was disrupted, reducing the content of undesirable linoleic acid while simultaneously increasing its desirable precursor, oleic acid. With the discovery of DNA-free CRISPR-Cas9 genome editing in plants, these same types of modifications could be made to other nutritive regulator genes in a much shorter timescale (Ma et al., 2020). While these systems are subject to less regulation, they are unable to incorporate genes in heterologous pathways, limiting their use in plant synthetic biology. In addition to regulatory concerns, criticism of genetically modified crops by the public has slowed the advancement of crop engineering. Further engagement between scientists and the public will be needed to improve the overall opinion of transgenic crops (Wurtzel et al., 2019).

### Delivery of Health-Promoting Small Molecules

While supplements generated through microbial production or extraction from a native plant host can often be used as a source for the consumption of nutrients, plants serve as both a production platform and a vector for the delivery of nutritional and therapeutic small molecules. In addition to removing the need for costly purification processes, the delivery of small molecules through edible plants provides a low-cost solution to delivering critical nutrients and medicines. The expression of pathways to improve the nutrient profile of staple crops is thus a promising method to improve access to the health benefits of a diverse diet. Anthocyanins are commonly consumed flavonoids with antioxidant activity that are believed to play a preventive role in many diseases, including cancer (Tsuda, 2012). With the expression of two transcription factors from *A. majus* (snapdragon) by a fruit-specific promoter, tomatoes, normally devoid of anthocyanins, accumulated high concentrations of 2.83 ± 0.46 mg/g fresh weight and displayed purple coloring. Subsequent feeding experiments in cancer-susceptible *Trp53−/−* mice with purple, anthocyanin-producing tomatoes significantly improve life span compared to control groups fed a normal diet or diet supplemented with red tomatoes (Butelli et al., 2008). If made available to the public, purple tomatoes could serve as a broadly available source of health-promoting anthocyanins. Additionally, anthocyanin-producing purple rice was developed through the expression of eight transgenes driven by endosperm-specific promoters, resulting in anthocyanin accumulation of 1 mg/g dry weight (Figure 1; Zhu et al., 2017). By generating these crops, nutraceuticals can be delivered through the foods we eat, rather than with the use of a supplement. This has the potential to improve the accessibility of nutraceuticals.

Artemisinin is a sesquiterpene lactone used in the treatment of malaria, whose biosynthesis has received a large amount of attention. One study optimized the production of artemisinin through the compartmentalization of the biosynthesic pathway to the cytosol, mitochondria, and chloroplast in *N. tabacum* (tobacco). Crushed tobacco leaves synthesizing artemisinin, wildtype tobacco leaves, and pure artemisinin were then fed to mice infected with *Plasmodium berghei*, and parasitemia was monitored. Mice fed with tobacco leaves containing artemisinin showed reduced parasitemia and delayed onset of malaria symptoms compared to mice fed wild type tissue and pure artemisinin (Malhotra et al., 2016). While tobacco is not considered an edible crop, this approach shows the potential for engineered foods to serve as a delivery vehicle for important therapeutics. As plant synthetic biology advances and the yields of therapeutic small molecules in plants are improved, developing methods to control the dose of the target molecule will be needed.

### Intra and Intercellular Compartmentalization

As organisms with multiple cellular compartments and tissue types, plants are ideally suited for the expression of complex metabolic pathways that require compartmentalization (Figure 1). The precise control over localization is particularly important when pathway intermediates are toxic. Strictosidine is an important intermediate in the production of the chemotherapy drug, vincristine. The strictosidine aglycone is a toxic intermediate in the strictosidine biosynthetic pathway that is normally sequestered to the vacuole before glycosylation. Viral induced gene silencing of a suspected strictosidine-glycosyl transporter, CfNPF2.9, resulted in blackening of leaf tissue in *C. roseus*, displaying the necessary role of transporters in plant natural product biosynthesis (Payne et al., 2017). As many metabolic pathways are compartmentalized, the use of transporters permits the movement of key intermediates synthesized in one compartment to the location of the enzymes required for the final steps of a biosynthetic pathway. This is further emphasized in the biosynthesis of aliphatic and indolic glucosinolates, which takes place in the cytosol and chloroplast. One study utilized a bile acid transporter to improve the heterologous yields of a precursor to the chemopreventive glucosinolate, glucoraphanin. Bile acid transporter 5 was demonstrated to be a plastidic transporter of the methionine-derived α-keto acid and increased the production of the glucoraphanin precursor, dihomomethionine, by 10-fold (Crocoll et al., 2016).

Many metabolites are synthesized in one tissue and transported to another as a means of storage or defense. Intercellular glucoraphanin transporters (GRT1 and GRT2) have been identified, which are responsible for the transport of glucoraphanin to seeds in *A. thaliana* (Nour-Eldin et al., 2012). The identification of these transporters allows for the transport of chemopreventive glucoraphanin to seeds of crops species to improve dietary consumption. Additionally, the use of transporters could allow for the concentration of metabolites into a specific tissue type, which would simplify harvest and extraction. Thus, there is a need to increase our understanding of transporters to enable the utilization of compartmentalization in the heterologous expression of metabolic pathways. However, most current methods are low throughput relying on the generation of complex yeast strains or plants with mutant transporters (Nour-Eldin and Halkier, 2013). One method utilizing *Xenopus laevis* oocytes expressing putative transporters has been demonstrated to improve the throughput of transporter screening by multiplexing transporters (Nour-Eldin et al., 2006, 2012). Moreover, there is also a severe lack of structural information on plant specialized metabolite transporters, with less than a handful of known structures (Miyauchi et al., 2017; Tanaka et al., 2017). An increase in the number of solved metabolite transporter structures could alleviate the need for enzymatic characterization through the use of prediction software, such as TransportTP (Li et al., 2009).

The compartmentalization of plant natural products into different tissue types provides a unique means for the production and storage of nutraceuticals and phytochemicals. Tissue-specific promoters enable the expression of metabolic pathways in a select tissue type. In one study, a bidirectional, embryo-specific promoter was used to drive the expression of anthocyanin genes in maize (Liu et al., 2018a). The bidirectional promoter was then engineered with additional *cis*-elements to generate a construct expressing anthocyanin genes in both the endosperm and embryo (Liu et al., 2018b). Additionally, specific plant tissues, such as trichomes, naturally accumulate large amounts of valuable plant natural products, thus making them promising targets for metabolic engineering. In one study, two genes required for methylketone synthesis, *ShMKS1* and *ShMKS2*, were expressed constitutively alone or together in *N. tabacum*, *A. thaliana*, and cultivated tomato (*S. lycopersicum)*. While this resulted in methylketone production, it also caused severe lesions. However, by expressing *ShMKS2* using a trichome-specific promoter, a significant increase in methylketone levels was observed with no other morphological defects (Yu and Pichersky, 2014). In another study, the trichomes of *G. hitsutum* L. (cotton) were engineered to produce melanin through the expression of two genes driven by a cotton fiber-specific promoter. This enabled the production of brown-colored cotton fibers containing melanin, which could serve as a UV protectant and a natural pigment (Xu et al., 2007). These studies show the efficacy of tissue-specific expression as a means to produce useful small molecules. Future research should aim to identify and develop tissue-specific promoters that allow for targeted expression of pathways.

Plant compartmentalization also enables organelles to produce and accumulate high amounts of a specific molecule of interest (Figure 1). Much work has been done to improve the availability of plant natural product precursors by targeting enzymes to specific organelles. Amorpha-4,11-diene is an important precursor to artemisinin. By targeting two enzymes involved in amorpha-4,11-diene synthesis to the chloroplast, amorpha-4,11-diene concentrations were improved by 40,000-fold compared to plants targeting the two enzymes to the cytosol (Wu et al., 2006). Additionally, the localization of enzymes can alter its stability or activity. One study found that targeting tryptophan decarboxylase to the chloroplast improved accumulation and stability of the enzyme compared to its cytosolic counterpart (Di Fiore et al., 2002). Targeting enzymes to different cellular compartments can substantially improve the function of enzymes and the production of plant natural products.

### Host Engineering

The expression of heterologous pathways can introduce a tremendous metabolic burden on the host organism. The use of large amounts of cellular resources, such as carbon sources, ATP, and NAD(P)H, can lead to undesirable physiological changes that reduce host fitness and product titers (Wu et al., 2016). Mitigating the metabolic burden of heterologous pathways can be accomplished through host engineering. Additionally, the use of engineered hosts can improve the yields of target metabolites.

Microbial synthetic biologists have used host engineering to improve the production of various important small molecules, which can serve as an example for plant synthetic biology. Phenylalanine is an important precursor for the synthesis of many plant natural products, such as phenylpropanoids and aromatic glucosinolates (Halkier and Gershenzon, 2006; Vogt, 2010) as well as many industrial applications, such as aspartame production (Bongaerts et al., 2001). The *E. coli* strain, ATCC31884, has been engineered to overproduce phenylalanine. This strain has enabled the high production of multiple molecules of interest. One study utilized ATCC31884 alongside changes to phenylalanine, tyrosine, and shikimate biosynthesis to improve the production of chorismate. The high chorismate-producing strain was then used to produce muconic acid, which resulted in yields of 1.5 g/L after 48 h of growth (Lin et al., 2014). ATCC31884 has been further altered to improve the diversity of chemicals it can be engineered to make. Huang et al. (2013) knocked down two genes required for phenylalanine expression in ATCC31884 while expressing a feedback-insensitive mutant of *tyrA* to improve the levels of tyrosine, which enabled the strain to make 15-fold greater caffeic acid than the previous highest yield (Huang et al., 2013). These examples of microbial host engineering should serve as inspiration for plant synthetic biologists to engineer plant production platforms for optimized production. Plants like *N. benthamiana* could be altered in a similar fashion to *E. coli* strain ATCC31884 as a plant background designed for the production of phenylalanine-derived plant natural products.

While select plants have been utilized for metabolic engineering, individual species have undergone limited host engineering. This highlights a current need for the field, as host engineering could be a valuable means to improve the yields and quality of plant-produced biomolecules. Plants are increasingly used for the production of antibodies; however, their use has been hindered due to differences in N-glycosylation patterns compared to mammalian cells (Strasser et al., 2008). Strasser et al. (2008) utilized host engineering to ameliorate this issue. Using RNAi, genes encoding a ß-1,2-xylosyltransferase and an α-1,3-fucosyltransferase were knocked down in *N. benthamiana*. This resulted in a strain of *N. benthamiana* capable of producing the antibody 2G12 with the same glycosylation as 2G12 produced in Chinese hamster ovary cells without noticeable effects on plant health (Strasser et al., 2008). Strains of soybean and *Arabidopsis* have also been engineered with the use of a feedback-insensitive variant of cystathionine gamma synthase, which leads to the accumulation of methionine (Matityahu et al., 2013; Song et al., 2013). These strains can serve as a platform for further engineering of nutraceuticals such as the methionine-derived glucosinolate, glucoraphanin. Additionally, improving the biomass of production platforms could further improve yields. One study generated tobacco plants expressing a synthetic glycolate pathway that improved biomass productivity by 19–37% (Figure 1; South et al., 2019). The use of this strain could improve the viability of plants as a production platform for therapeutic small molecules. Host engineering could prove to be a powerful tool for plant synthetic biologists to push the boundaries of their production platform.

## Conclusion

Plant synthetic biology and metabolic engineering have the ability to tailor crops for specific nutritional and medicinal needs. As we have highlighted, there are many ways in which plant biologists have already enhanced crops through biofortification to improve macro- and micronutrient consumption. Though, there is still room for improvement. Plant specialized metabolites are regularly used for medicinal purposes. Plant synthetic biology can enhance the production of bioactive compounds especially when the native host plants are difficult to cultivate, or the concentration of bioactive compounds are too low for purification. Additionally, compounds with disease preventive capacity can be expressed and accumulated in food crops, providing a cheap and effective strategy for nutraceutical delivery. As more pathways are discovered and compound efficacies are tested, the potential for food enhancement options will continue to grow, allowing us to further expand the boundaries of future tailored crops.

## Author Contributions

CB and BE contributed equally to preparation of this work. CB, BE, and PS contributed to the editing and discussion of the manuscript. All authors contributed to the article and approved the submitted version.

## Conflict of Interest

The authors declare that the research was conducted in the absence of any commercial or financial relationships that could be construed as a potential conflict of interest.

## Publisher’s Note

All claims expressed in this article are solely those of the authors and do not necessarily represent those of their affiliated organizations, or those of the publisher, the editors and the reviewers. Any product that may be evaluated in this article, or claim that may be made by its manufacturer, is not guaranteed or endorsed by the publisher.
